# An AI-mediated framework for recursive learning: transforming individual experiences into organizational knowledge and autonomous engagement in elderly care

**DOI:** 10.3389/fdgth.2025.1529072

**Published:** 2025-07-03

**Authors:** Naoki Mitsugi, Koki Ijuin, Chiaki Oshiyama, Eri Hara, Takuichi Nishimura

**Affiliations:** ^1^ School of Knowledge Science, Japan Advanced Institute of Science and Technology, Nomi, Japan; ^2^ Research and Development Division, C-Cube Inc., Arakawa, Japan

**Keywords:** elderly care, AI-assisted, experiential learning, organizational learning, reflective practice, knowledge sharing, daycare service

## Abstract

**Introduction:**

The elderly care industry in Japan is currently facing significant challenges, including chronic labor shortages, high staff turnover, and low levels of IT utilization. Effective human resource development is crucial to address these issues.

**Methods:**

In this study, we propose an AI-supported method to facilitate both individual and organizational development in elderly care settings. This approach integrates Kolb's experiential learning theory and Huber's organizational learning framework. Staff members use digital tools to record their daily observations, which are then processed by an AI system. The AI organizes and visualizes the data to promote structured reflection and behavioral improvement.

**Results:**

The visualized data serve as a basis for personalized guidance provided by managers and senior staff, tailored to each employee's personality and situation. This process fosters cooperation and knowledge sharing within the organization. The demonstration study showed that AI-supported reflection enhanced organizational collaboration, improved work procedures, and increased staff's goal awareness and proactive behavior.

**Discussion:**

Furthermore, individual experiences were transformed into structured, purpose-oriented organizational knowledge through AI-driven analysis and were utilized as manuals. The method ensures cost efficiency and adaptability for small-scale care facilities through the use of digital infrastructure. It provides a practical and scalable solution to enhance care quality and workforce development.

## 1 Introduction

“In Japan, with the aging of the population and declining birthrate, the shortage of personnel and increased workload for staff in the elderly care industry have become serious issues.” (1). In other words, Japan is facing serious issues of labor shortages and increased workloads for staff in the elderly care industry. In 2021, the population aged 65 and older was 2.45 times that of children under 15. This is the highest aging index in the world, far exceeding Italy's 1.85, and more than 20 times that of Bangladesh's 0.1, indicating a very serious situation.

This makes the situation extremely critical. This means that in Japan, the working population will be extremely small compared to the elderly population in the future (1). As a result, the burden on elderly care staff is increasing year by year, and according to statistics from 2021, 70% of the elderly prefer to be cared for at home, and the number of elderly who prefer to be cared for at home is approximately four people per elderly home care worker. Elderly care staff are responsible for a variety of tasks, including supporting the elderly in their daily lives and providing physical assistance to those who are unable to move around by themselves. Moreover, they work in a physically and mentally demanding environment and are paid lower wages compared to other jobs in the same age group. As a result, the turnover rate is high, and it is not easy to secure and maintain elderly care staff (1).

Since the 2000s, robots and AI pets have been increasingly adopted in nursing care settings. The introduction of nursing care support robots and AI-powered administrative support tools has led to efforts to reduce the physical burden on elderly care staff and improve work efficiency (2, 3). In recent years, since 2020, AI has been increasingly used to predict health behaviors and support decision-making. For example, predictive models combining AI and IoT are being developed to monitor the behavior and health status of the elderly and enable individualized responses (4). AI-supported decision-making systems (AI-DSS) for long-term care are also being introduced. These care robots, AI pets, and AI-based task assistance tools are expected to reduce the burden on frontline staff.

There is also an approach to solving the elderly care issues in Japan through the development of the elderly care workers and the organization. When workers feel that they can develop their capabilities through their work activities and gain a sense of self-efficacy through their knowledge and day-to-day work, it leads to better work efficiency and staff retention (5). However, the current situation is that elderly care workers have few opportunities in which they can feel that they are “learning” or “developing their skills” through their daily work activities, and in many cases, their motivation and enthusiasm are declining (6, 7). In addition, knowledge tends to stay within the minds of individual workers in the care workplace, and individual experiences do not necessarily contribute to quality improvement across the organization (8). If we are to address the problem of a severe shortage of workers, we should support the development of those currently engaged in elderly care and develop “human resources” who can perform their duties with a deeper insight. Using AI, we need to create an environment where effective knowledge-sharing methods are used and quality improvement of both individuals and the organization is carried out continuously. In other words, the best way to reduce the burden on elderly care workers is to enhance the capabilities of each care worker, share knowledge within the organization, improve the organization as a whole, and enable highly effective mutual support.

Zheng et al. (9) reported that AI-based feedback for improving behavior in online learning communities for university students could improve collaborative learning outcomes. It has been confirmed that AI can support individual knowledge construction, enhance collaborative processes, and promote organizational performance and collaboration. There is a need for a method of supporting learning using AI that can also be implemented in the elderly care field, including elderly care.

This study proposes a method for supporting learning that can be applied to interpersonal services and enables a learning cycle for individuals and organizations. In particular, we propose a method for supporting learning that utilizes AI to improve objectivity and comprehensiveness.

Kolb (10) advocates that experiential learning promotes effective learning through four steps: concrete experience, reflective observation, abstract conceptualization, and active practice. When this theory is applied to elderly care workers, they can reflect on their daily experiences and put their new insights into practice. In addition, in Huber's organizational learning, knowledge is accumulated in an organization through four steps: knowledge acquisition, information distribution, information interpretation, and organizational memory, thereby improving the organization's ability and adaptability (11).

This study aims to promote learning and organizational growth in elderly care by integrating individual experiential learning and organizational learning and proposing a mechanism for AI-enabled feedback and knowledge sharing. By doing so, we intend to clarify the impact of AI-enabled knowledge sharing and structuring on individual learning and organizational growth, contribute to the creation of academic knowledge, and resolve practical issues such as business efficiency and staff motivation.

## 2 Related research

### 2.1 Implementation of technology in elderly care

Since the 2000s, care robots and AI pets have been introduced in the field of elderly care. These are expected to reduce the physical burden on staff and contribute to improved work efficiency (2, 3). For example, Wada and Shibata (2) demonstrated that “Paro,” an AI-equipped pet-type therapy robot, has a positive effect on the psychological stability of elderly people. Additionally, Wada et al. (24) reported that robot therapy is effective in reducing stress among the elderly. On the other hand, while the introduction of elderly care robots continues, Yamada (3) points out the issues related to the acceptance of technology in the field and the limitations to practicality elderly.

Furthermore, in recent years, since 2020, new technologies such as AI-based prediction of health behaviors and decision support have been introduced in addition to these. For example, AI and IoT-combined predictive models enable real-time monitoring of elderly individuals' behavior and health status, facilitating personalized responses (4). Additionally, AI-supported decision-making systems (AI-DSS) for long-term care are being implemented, and determining how much trust to place in AI's judgments and the division of roles between humans and AI are critical challenges in care settings (12).

In addition, Broadbent et al. (13) found that the acceptance of technology in the elderly care field depends on culture and social background and stated that it is important to consider the field's characteristics when introducing technology. These studies show that while the introduction of technology contributes to improving work efficiency, there are many issues related to its acceptance and operation.

While the introduction of technology contributes to improving operational efficiency, it should be utilized not as a replacement for humans but as a tool to assist in their work.

### 2.2 AI assisted education

In a study by Zheng et al. (9), it was reported that the use of AI in education has the potential to promote collaborative learning. It was also shown that real-time feedback and guidelines for improving behavior support the development of learners. This finding describes the effectiveness of AI-assisted reflection in education but does not mention its application in elderly care.

In addition, the study by Holmes et al. (14) shows the effectiveness of AI-based learning support systems in promoting individual and organizational learning. Baker and Siemens (15) point out the effectiveness of data-driven learning through the fusion of learning analytics and AI technology. These findings form the basis for new AI methods in designing staff education programs in the elderly care field.

### 2.3 Promoting knowledge sharing and issues

In interpersonal services such as elderly care, knowledge sharing among staff members is known to be an important issue. According to Senge (8), knowledge sharing is a core element of organizational learning, and converting individual knowledge into organizational learning enables the improvement of overall organizational capability. In addition, Nonaka and Takeuchi's (16) SECI model systematically shows the process of converting tacit knowledge into explicit knowledge and sharing and utilizing knowledge. This provides a framework for knowledge to be effectively shared between individuals.

Davenport and Prusak (17) pointed out that knowledge sharing is important in increasing an organization's competitiveness. Argote and Miron-Spektor (18) stated that organizational knowledge is generated through the process of converting experience into knowledge and that this process plays a central role in organizational learning. These studies show that knowledge sharing is the foundation for promoting growth throughout the organization.

On the other hand, Ijuin et al. (19) proposed a method for clarifying the purpose of each action within the work procedures in elderly care settings and constructing a “purpose-oriented knowledge graph” based on this. This study presents a new framework for promoting knowledge sharing by structuring the purpose of each action, particularly in specific elderly care tasks such as feeding and toileting assistance. However, the practical application of knowledge structuring requires the cooperation of experts, and there are still issues to be addressed in applying the knowledge that is updated on a daily basis at the workplace to structured knowledge by the workers themselves.

### 2.4 The effectiveness of applying learning theory to the elderly care workplace

Senge's (8) organizational learning presents an abstract framework in which the entire organization builds a culture of continuous learning and sustainable growth by responding flexibly to change. On the other hand, Kolb's (10) experiential learning explains how individuals gain awareness and develop capabilities through work activities in four steps: concrete experience, reflective observation, abstract conceptualization, and active practice. Huber (11) also developed a concrete method for making knowledge of individuals and organizations available to the entire organization through four processes: knowledge acquisition, information distribution, information interpretation, and organizational memory. Furthermore, Bandura (5) shows that self-efficacy is the psychological foundation that promotes individual learning and behavior through belief in goal achievement.

Although these theories provide a practical framework and method for supporting individual and organizational learning, the following issues remain in adapting them to interpersonal services such as elderly care. Kolb's experiential learning is effective for individual learning but lacks a mechanism for linking it to organizational growth. On the other hand, while Huber's method provides a practical knowledge-sharing framework, it does not directly support individual growth or motivation. In addition, while Senge's organizational learning is theoretically plausible, it is necessary to incorporate it into a specific methodology to put it into practice. Furthermore, while Bandura's self-efficacy is an important element that supports the behavior of individual staff members, a system that integrates individual awareness into goal-oriented experiential learning and feeds it back to the team and organization has not been thoroughly considered.

In this study, we will integrate these theories and methods to create a practical model that effectively links individual learning and knowledge sharing across the organization and then verifies its effectiveness in elderly care.

## 3 Proposal for a method of cyclical individual and organizational learning utilizing AI

In this chapter, we propose a mechanism for using AI to support individual experiential learning and organizational learning in the field of elderly care, thereby creating a cycle of learning. We explain specific methods and their constituent elements.

### 3.1 Proposed method: A method for cyclical individual and organizational learning utilizing AI

We propose a new learning method that integrates individual experiential learning and group organizational learning. We will apply this method to the interpersonal service field of elderly care. More specifically, we will design a system that links Kolb's (10) experiential learning and Huber's (11) organizational learning with AI to maintain objectivity and comprehensiveness and creates a cycle of individual growth and organizational knowledge sharing.

**Figure 1:**
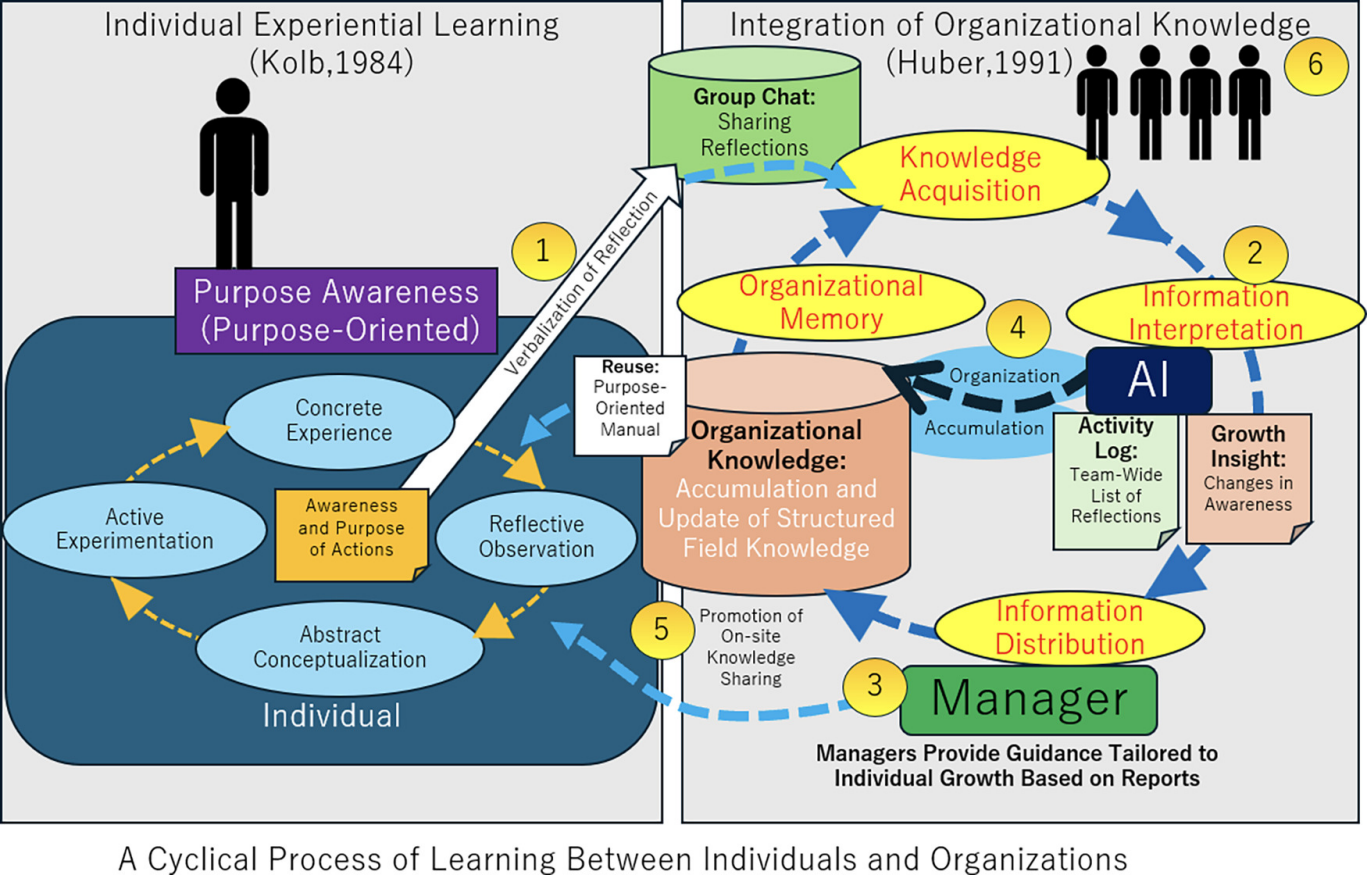
Cycle of organizational learning through individual reflection.

As shown in Figure 1, in the proposed method, staff first reflect on their daily work and put their thoughts into words. The resulting text, consisting of approximately 100 characters and including insights and the purpose of each action, is posted to a group chat and sent to AI as data. The AI then analyzes the individual reflections and behavioral trends and visualizes them in text form. Using this AI-based list and analysis results, we will promote staff learning and build a system to promote further learning through knowledge sharing within the organization. The aim of the proposed method is to achieve both individual development and enhancement of organizational work at the same time. Providing an environment where individual staff members can enhance their learning while maintaining a sense of purpose through their work activities also promotes knowledge sharing and integration throughout the organization. This mechanism is expected to improve the quality of work and ensure the organization's sustainable growth.

The following shows the AI-enabled learning cycle process for individuals and organizations proposed in this study. Table 1 summarizes each process, and Figure 1 provides a visual overview of the entire process.

**Table 1:** The process from individual reflection to organizational learning.

Step	Contents
1. Individual Reflection (Number 1 in the diagram)	Perform daily tasks with a sense of purpose and look back daily to proactively discover (awareness and the purpose of the action). Document reflective observations and abstract conceptualizations as reflections. Verbalization of Reflection serves as the gateway to Knowledge Acquisition for the organization.
2. Objective analysis by AI (Number 2 in the diagram)	AI performs analysis and reporting based on the reflective data; AI generates a list of everyone's reflections for a week and analyzes trends in changes in individual awareness from a month's worth of data. This refers to Information Interpretation by AI.
3. Development support by management (Number 3 in the diagram)	Through daily operations, managers will provide guidance based on AI analysis results and make appropriate interventions to enhance individual learning and support development. This refers to Information Distribution by the Manager.
4. Sharing learnings within the organization (Number 4 in the diagram)	Through the sharing of daily work, awareness, and purpose-oriented manuals, the learning of participating members is shared throughout the organization. Sharing facilitates the circulation of knowledge and experience, and extracts the common purpose of the organization. Along with this, it strengthens the individual's sense of purpose. This refers to On-site Knowledge Sharing.
5. Structuring knowledge by AI (Number 5 in the diagram)	AI structures the knowledge related to the work from the reflective data and stores it as organizational knowledge (purpose-oriented manual) that can be shared throughout the organization. This refers to the construction of Organizational Memory by AI.
6. Integration of organizational work and knowledge (Number 6 in the diagram)	Across all steps from 1 to 5, individual reflection becomes Knowledge Acquisition and is accumulated as Organizational Memory. Utilizing organizational memory in daily operations strengthens organizational coordination and improves the efficiency of collaborative work.

### 3.2 Individual reflection

In Step 1, individual staff members reflect on the “concrete experiences” in their daily work activities and record their insights and the purpose of their actions. This process utilizes the steps of experiential learning and proceeds through the following four stages:

Concrete Experience: Staff members' experiences in their work activities in the field form the starting point for learning.

Reflective Observation: Staff members reflect on their experiences and consider the insights gained and the context of their actions.

Abstract Conceptualization: Staff members summarize their experiences and transform them into abstract knowledge and concepts that can be applied.

Active Experimentation: Staff members try out new behaviors based on abstract knowledge, which improves their fieldwork and leads to personal development.

By going through these four stages, staff members can strengthen their sense of purpose, increase their understanding of their work, and lay the foundations for improving the quality of their subsequent actions (10, 11). Next, by recording the insights and learning gained through reflection and sharing them in group chats, individual learning is shared and utilized as organizational knowledge, creating an environment that promotes mutual growth.

### 3.3 Data collection and organization using AI

In Step 2, AI collects reflection data recorded by staff members using a designated text input form to post in group chats and then organizes and visualizes that data. This data is summarized in a format that can be shared among all staff members, listing individual insights and the purposes of their actions. Furthermore, the AI performs trend analysis based on this data to clarify the progress and challenges of learning for both individuals and the organization. This information is used by managers for guidance, serving as a foundation for data-driven guidance. Through AI analysis, guidance can be implemented tailored to each staff member's individual circumstances and the organization's overall challenges.

This visualization is not in the form of graphs or charts but rather descriptive summaries of reflection records through AI natural language processing. For example, monthly reports include descriptions such as “Improvement proposals were made based on user reactions during recreational activities” and “Support that respects user autonomy was observed,” indicating individual staff members’ learning trends and behavioral changes.

**Figure 3:**
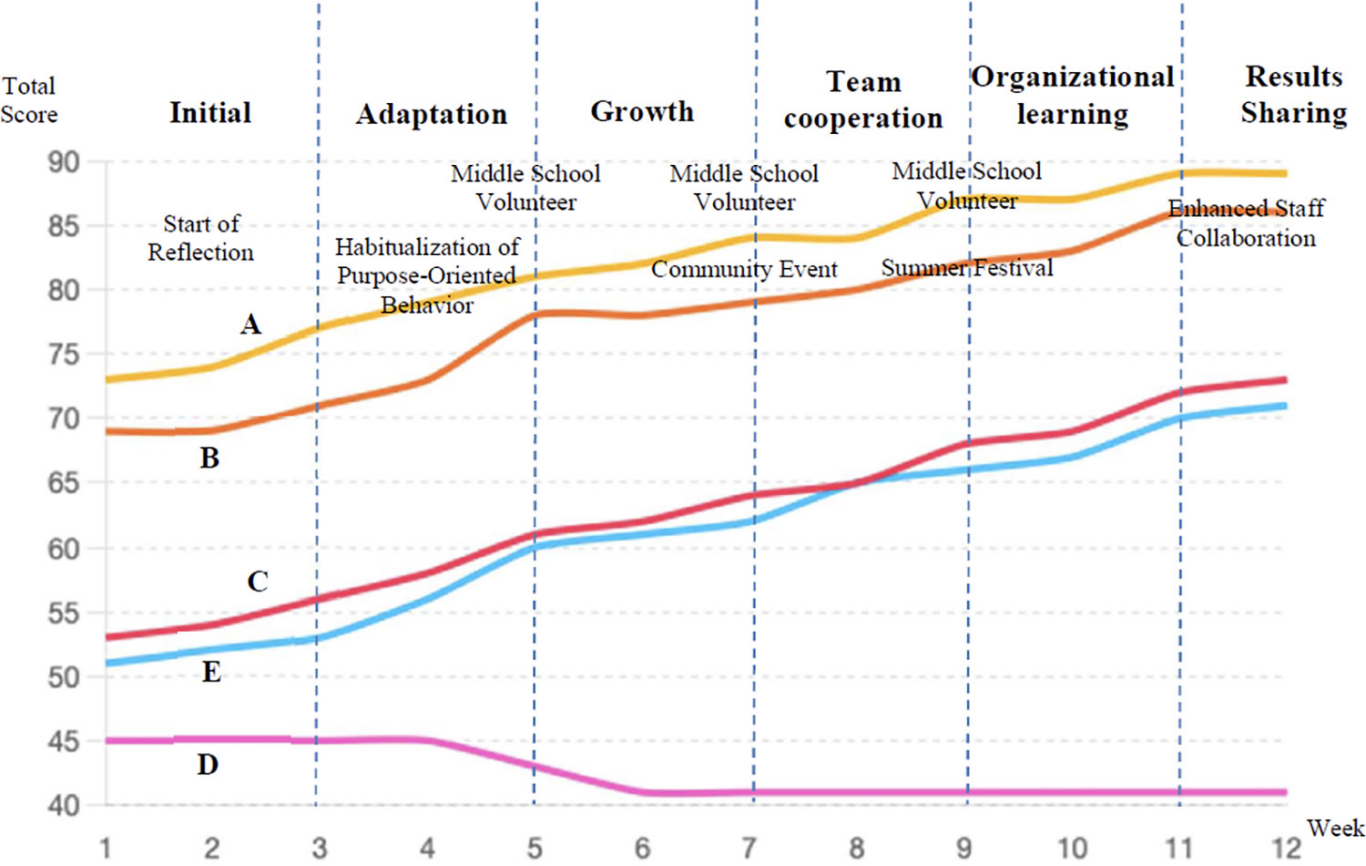
Individual evaluations over time.

Additionally, weekly reports organize individual staff insights and their objectives in tabular form, which are utilized for managerial guidance and team-wide learning sharing. Examples of these outputs are shown in Tables 2 (weekly) and 3 (monthly) in Chapter 4, with the visualization structure illustrated in Figure 3 and the natural language processing functions and output formats used detailed in Table 4.

**Table 2:** Summary of periods in study.

Period	Phase	Description
Weeks 1–2	Introduction to reflection	Through the introduction of reflection, staff showed interest in autonomy and a sense of purpose. Through the reflections, staff became aware of the changes and began to actively work on improving their work.
Weeks 3–4	Habituation and awareness	Daily reflections became a habit, and an awareness of the need to improve operations began to appear in their actions. Depending on the situation, they were now able to flexibly change procedures to achieve their objectives.
Weeks 5–6	Learning enhancement phase	Staff problem-solving skills improved and leadership skills began to emerge; managers were guided by AI reports and showed a commitment to improving behavior.
Weeks 7–8	Organizational cooperation strengthening	Collaboration among staff members increased and collaboration was expressed through events such as summer festivals. Organizational work scores improved significantly, and organizational change was evident in event activities.
Weeks 9–10	Organizational learning phase	Ongoing changes in the organization have been identified and the quality of user support has improved. A culture of interest in/teaching of other staff reflections in group chats was developed.
Weeks 11–12	Result-sharing phase	Collaboration throughout the organization was strengthened, leading to actions in line with the facility's philosophy; regular reflections through AI reports took root, and everyone in the organization shared a sense of purpose.

**Table 3:** Effect sizes and confidence intervals for staff development scores.

Evaluation item	Mean before	Mean after	Cohen's *d*	95% Confidence interval
Independence	5.4	7.4	1.03	−0.29 to 2.34
Problem-solving ability	5.8	7.2	1.07	−0.25 to 2.40
Awareness of business improvement	5.4	7.2	1.09	−0.24 to 2.41
Organizational work	6.6	7.6	0.57	−0.69 to 1.84
Leadership	5	6	0.58	−0.69 to 1.84
Sense of responsibility	5.4	7	1.03	−0.29 to 2.35
Capacity for empathy	5.6	7.4	1.08	−0.25 to 2.40
Flexibility	5.4	7.2	1.2	−0.15 to 2.55
Continuity	5.8	7	0.68	−0.59 to 1.96
Non-egocentrism	6.4	7.4	0.75	−0.54 to 2.03

**Table 4:** NLP features used in AI generated reports.

Classification	Weekly reports (listings)	Monthly reports (describing extracted changing trends)	Objective-oriented manual generation
Data extraction	Named entity recognition (NER) (extract date, staff name, content) sentence segmentation (isolate sentences)	Named entity recognition (NER) (extracts data over multiple weeks)	Named entity recognition (NER) (extract roles, times, procedures, and purposes)
Data classification	Text classification (classify “Awareness” and “Purpose”)	Text classification (data classified on a weekly basis and consolidated on a monthly basis)	Text classification (organize information by role and purpose)
Frequency analysis	Keyword extraction (extract important words from individual “observations” and “objectives”)	Frequency analysis (aggregate frequency of occurrence of important keywords over multiple weeks)	Frequency analysis (identify words that appear repeatedly in objectives and work procedures)
Trend analysis	Not required (trend analysis is not performed on weekly reports)	Topic modeling (extract frequently occurring topics) Emotional analysis (identify positive/negative changes)	Topic modeling (organizing key themes based on roles and objectives)
Time series analysis	Time series analysis (data sorted by date and aggregated weekly)	Time-series analysis (weekly changes are aggregated on a monthly basis to visualize trends)	Time series analysis (organize work procedures in chronological order to show efficient flow)
Data structuring	Conversion to tabular format (organize dates, staff names, observations, and objectives)	Consolidation of analysis results (organized by trend/topic)	Structuring data by role, purpose, and time series (integrate procedures, objectives, and vision)
Output format	Table format (markdown or CSV/excel)	Natural language generation (NLG) (generate the results of the analysis as explanatory text)	Natural language generation (NLG) (generated in tabular form with roles, procedures, objectives, and vision)

### 3.4 Manager-led growth support

In Step 3, managers use the reports provided by AI to provide guidance that promotes staff development. This guidance is provided on site as needed, for example, by providing specific solutions to issues that arise during work activities. In addition, managers directly interact with staff members one-on-one, providing guidance and feedback tailored to each individual's development. Through this individual guidance, staff members will be able to enhance their learning and receive support to improve their behavior at the workplace.

### 3.5 Structuring knowledge with AI

In Step 4, AI structures knowledge in a goal-oriented manner based on reflective data and shared learning. This structured knowledge is accumulated as dynamic data and used in new employee training and monthly meetings. This makes implicit knowledge in the workplace explicit and puts it in a form that can be used as practical knowledge. This accumulation of knowledge is not just an update of manuals but an important asset that supports decision-making and business improvement in daily operations.

### 3.6 Sharing learning within the organization

In Step 5, the staff's reflections and the AI analysis results are shared throughout the organization. This process utilizes organizational learning. Through the four processes of “knowledge acquisition,” “information distribution,” “information interpretation,” and “organizational memory,” the knowledge interaction that occurs when a staff member shares his/her individual learning through group chats and weekly meetings creates a synergistic effect on learning. This sharing clarifies the organization's common goals and further strengthens each individual's sense of purpose. The organizational learning process improves the organization's knowledge base as a whole and promotes collaboration between members.

### 3.7 Integrating organizational work and knowledge

In Step 6, structured knowledge is used daily, strengthening collaboration across the organization. Through this process, individual learning is integrated as organizational knowledge, and the quality of problem-solving and decision-making onsite is improved. In addition, this knowledge is accumulated in the organization as a whole, and the learning cycle is continuously promoted. This enables growth across the organization and improves productivity and the quality of care services onsite. Incorporating organizational learning creates an environment where knowledge can be shared and utilized smoothly as an organization.

## 4 Research and results

This chapter describes the experimental setup, data collection methods, analysis techniques, and results obtained in this study. This chapter is divided into two sections. Section 4.1 describes the experimental environment, definitions of terms, data collection and analysis methods, AI processing details, prerequisites, and an overview of the facilities and participants involved in the study. Section 4.2 presents quantitative and qualitative evidence of changes facilitated by AI support and manager guidance and provides a detailed report on the specific change processes and outcomes.

### 4.1 Research setting

#### 4.1.1 Definition of terms used in this research

The definitions of the terms used in this research are as follows:

Reflection: After completing work activities, individuals write down their “awareness” and “purpose of the action.” This activity recalls memories, organizes information, and extracts the purpose of actions.

Group chat: LINE, Japan's most popular social media platform, was used as the group chat tool for staff to share daily reflections. In the facility, staff primarily used their personal mobile phones to access the LINE group chat. While desktop computers were available for administrative tasks, reflection sharing was conducted via mobile devices for convenience and immediacy.

AI tool: ChatGPT4o. Input daily staff reflections into a chat tool that converts them into text. Generate weekly activity reports and monthly growth analysis reports. Ensure that all input and output content does not contain personal information. Selected for its deep contextual understanding, ability to accurately grasp learning trends and intentions, stable operational foundation, and reliability.

AI report: The Activity Log is a list organized by AI based on the staff's reflections, and in this experiment, it is created weekly as a “Weekly Report.” The Growth Insight is a text report summarizing changes in individual awareness, and in this study, it is created monthly as a “Monthly Report.”

Purpose oriented manual: Data that summarizes the actions and purposes of staff reflections in the elderly care field and arranges them in chronological order for each work procedure.

Manager: Daycare service facility manager, organizational leader, manager.

Consultants: Daycare service consultants, sub-leaders of the organization, and managers.

#### 4.1.2 Data collection and analysis methods

Data collection methods involved collecting daily reflection records posted by staff members in a LINE group as data. AI-based analysis and outputs include: (1) Activity records (lists of reflections, weekly reports), (2) Growth analysis (analysis of trends in individual reflection content, monthly reports), and (3) Purpose-oriented manuals.

Additionally, researchers interviewed managers once a week outside of working hours, recorded the facility and staff conditions, and transcribed the interviews. Furthermore, 1 month after the experiment ended, feedback interviews were conducted with staff members, and the results were similarly recorded and transcribed.

The analysis methods are as follows. First, as a qualitative analysis, staff reflection records and manager interviews were classified based on the Grounded Theory Approach (GTA) (20), and factors contributing to insights and behavioral improvements were extracted. Next, for quantitative analysis, we set 10 evaluation criteria, as shown in Table 5, to measure staff growth and evaluated each item on a scale of 1–10. The scores are defined as follows:

1–3 points: Not achieved, or lack of behavioral change or purpose.
4–6 points: Some behavioral or awareness changes are observed, but there are issues with continuity and stability.
7–8 points: A stage where purposeful awareness is formed and voluntary actions are observed.
9–10 points: A stage where actions are established and can be shared with and guided by others.

**Table 5:** Evaluation items used in this study.

Evaluation item	TWCT items	Description
Independence	Initiative and goal setting	Ability to be proactive in meeting organizational goals.
Problem-solving ability	Collaborative problem-solving	Ability to work with organizational members to analyze issues and find solutions.
Awareness of business improvement	Task management and adjustment	Ability to improve operational efficiency and adjust processes as needed.
Organizational work	Inclusion in communication and collaboration	Smooth communication and mutual support within the organization.
Leadership	Task coordination and accountability	Ability to lead the organization and coordinate members to achieve goals.
Sense of responsibility	Accountability and task commitment	Ability to take responsibility for and ensure execution of assigned tasks.
Capacity for empathy	Included in interpersonal skills	Ability to understand and show empathy for the feelings and needs of organizational members.
Flexibility	Adaptability	Ability to adapt to changing circumstances and reallocate tasks appropriately.
Continuity	Commitment to team goals	Ability to continue to work toward long-term goals and cope with difficulties.
Non-egocentrism	Conflict resolution and collaboration	Ability to prioritize the interests of the organization as a whole and resolve conflicts constructively.

The evaluation criteria are based on the five core competencies of the “Teamwork Competency Test (TWCT)” developed by Stevens and Campion (21): conflict resolution, collaborative problem-solving, communication, goal setting and performance management, and planning and task coordination. Each item was reviewed by care professionals and verified using field data to ensure that it was consistent with the competency areas of the TWCT. As a result, the evaluation criteria were found to have scientific validity and applicability to the care setting.

Furthermore, due to the small sample size of five cases (*n* = 5), statistical significance tests were not conducted. Instead, effect sizes (Cohen's *d*) and 95% confidence intervals (CI) were calculated and presented in Table 3. This approach compensates for the limitations of the sample size and provides additional context for interpreting changes.

This study was designed as a theory-building field study using mixed methods to evaluate the practical application and theoretically examine AI-supported learning cycles in care settings. The analysis focused on descriptive trends, considering differences between AI outputs and human evaluations, to explore initial patterns in staff development.

#### 4.1.3 Data processing method using AI

Data processing is performed using ChatGPT-4.0, applying natural language processing (NLP) techniques tailored to the different outputs generated in this study: weekly reports, monthly reports, and purpose-oriented manuals (as shown in Table 4).

Although the neural network architecture is not customized, the processing is customized via prompt design and output format control. The NLP functions applied include named entity recognition (NER), text classification, frequency analysis, topic modeling, time-series analysis, and natural language generation (NLG). These functions enable the multifaceted analysis and structuring of staff reflection records.

This process extracts insights such as behavioral trends, shifts in purpose awareness, and knowledge to be shared within the organization. These outputs support knowledge sharing, staff guidance, and organizational learning.

**Figure 2:**
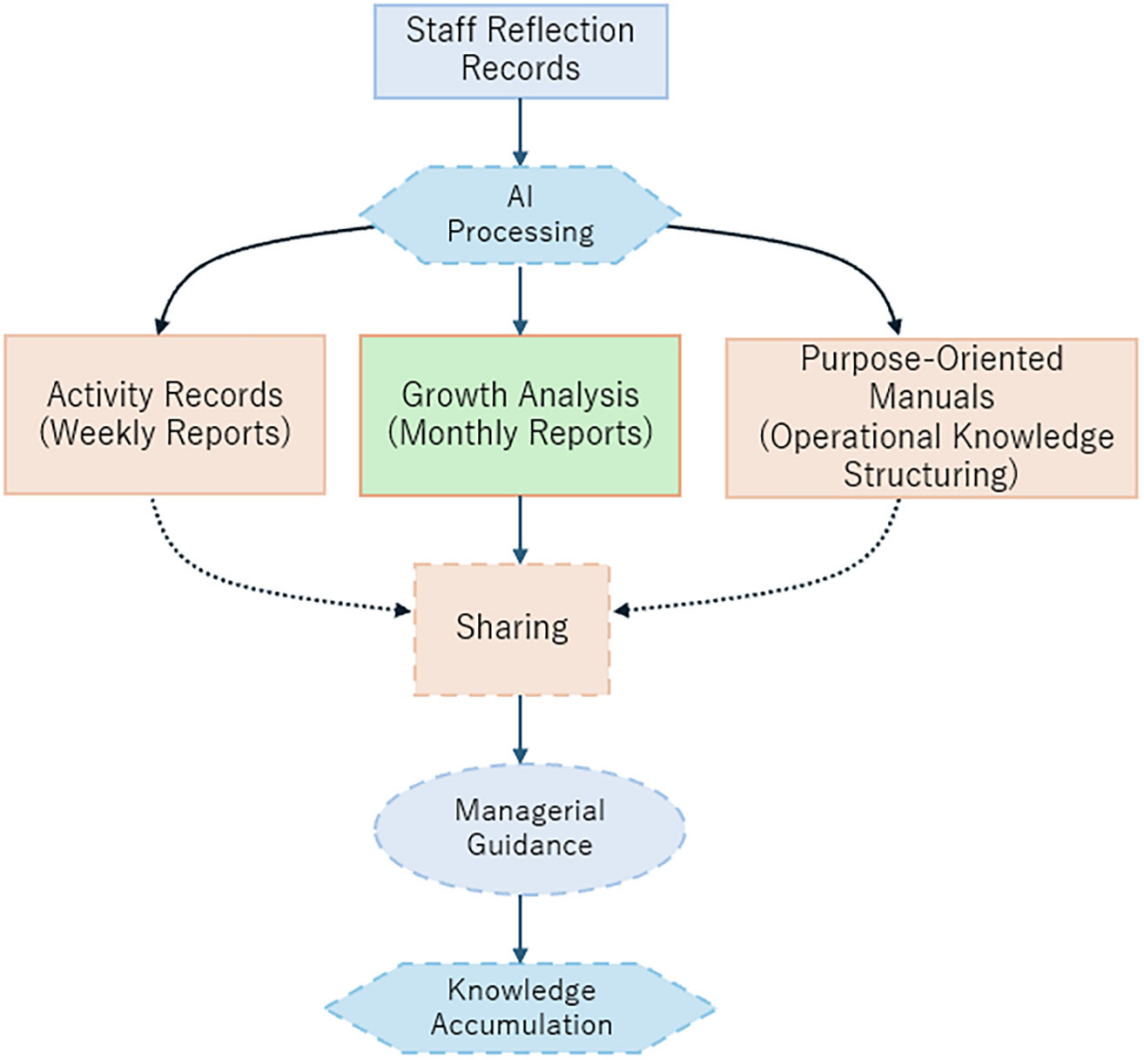
Overview of AI processing flow for reflection data and output generation.

Figure 2 illustrates the overall data processing flow. Staff reflection records are analyzed by AI and converted into three outputs: weekly reports (to capture overall trends), monthly reports (to track individual changes), and purpose-oriented manuals (to structure operational knowledge). The organization shares these outputs, supporting learning facilitation and knowledge accumulation.

ChatGPT-4o is a large language model (LLM) based on the Transformer architecture, using billions of parameters to process text context, syntax, and meaning. This study analyzes reflection records by identifying perspectives, evaluative words, and introspective phrases (e.g., “noticed that” “thought that… is important”) and summarizes their changes over time. It also captures word relationships and grammatical structures in conversations to generate summaries that reflect shifts in perspective (self → others → team). These features enable contextual analysis of reflection tendencies beyond simple keyword extraction. The specific processing functions are shown in Table 6.

**Table 6:** Processing functions and purposes of ChatGPT for reflection records.

Processing function (estimated mechanism within ChatGPT)	Description	Role in reflection analysis
Sequential context processing (tokenization and attention)	Decomposes input text into tokens and captures relationships within the context using self-attention.	Understands the overall meaning of text and weighs statements within their surrounding context.
Extraction of syntactic and pragmatic features	Identifies structures such as subject-verb relations, particles, evaluative words, and demonstratives to understand “who is thinking what.”	Contributes to detecting shifts in perspective (self/others/team) and identifying introspective expressions.
Detection of introspective expressions	Captures self-reflective phrases such as “noticed that” or “thought that… is necessary.”	Provides a basis for extracting key statements that indicate learning and growth.
Understanding semantic trends and changes in vocabulary	Tracks contextual changes in key terms over time (e.g., “hydration” → “maintaining health”).	Recognizes signs of deeper awareness or alignment with team goals.
Detection of perspective shifts	Detects shifts in focus from first-person to third-person or team-wide perspectives.	Captures the transition from individual reflection to organizational learning.
Abstractive summarization	Extracts key topics and reconstructs the context to present them in new expressions.	Presents staff's trends and changes in an understandable format, such as monthly reports.
Implication of emotional tendencies (implicit)	Does not use explicit emotion scoring, but reflects positive/negative expressions in the output.	May suggest elements of motivation or emotional change.

#### 4.1.4 Exploratory observation on the impact of AI support

In this study, we conducted an exploratory observation in a practical setting to examine how AI-generated reports affect work efficiency and changes in staff awareness. Due to operational constraints and the priority on real-world practice, rigorous comparative testing or the use of control groups was not implemented. We limited our observations to simple checks on how AI outputs influenced managers' ability to grasp record trends and changes in staff's descriptions of “awareness” and “objectives.” Although we conducted trial comparisons with other LLMs, such as Claude and Grok, these were not performed in a systematic manner. Further structured comparative analysis with different LLMs is planned for future research.

#### 4.1.5 Prerequisites for implementation

The following are the prerequisites for implementing this proposed method.

##### 4.1.5.1 Development of a digital environment

The introduction of group chat tools such as LINE is a prerequisite for staff to be able to reflect on their work activities on a daily basis. Developing an environment that allows staff to easily record their insights is essential.

##### 4.1.5.2 Data anonymity and privacy protection

The content of staff reflections and AI reports is anonymized to ensure thorough privacy protection. This helps create an environment where staff can record and share data with confidence and contributes to building trusting relationships.

##### 4.1.5.3 Active involvement of managers

To support staff development, managers are required to provide appropriate guidance through monthly reports based on staff reflections and AI report content.

##### 4.1.5.4 Accuracy of AI analysis

The effectiveness of AI reports is largely dependent on the quality of the staff reflections collected, so making the content of daily reflections more specific is necessary to improve their accuracy and usability.

Based on the above assumptions and preconditions, staff learning will progress cyclically, and staff development and learning throughout the organization will be promoted. In addition, by appropriately utilizing AI reports, managers can support staff development and knowledge sharing within the organization and contribute to improving the organization's overall performance.

#### 4.1.6 Overview of the facility monitored in the study

This study was conducted on elderly care staff at Daycare Service Facility P in Tokyo. Facility P opened in 2018 and has an average of 20 daily and 40 registered users. The facility's philosophy is to enable users to be “active for life” and provide “support for their independence.” In addition, in the daily work activities of elderly care, the facility contributes to the local community by providing a place for intergenerational exchange, such as accepting residents and student volunteers, using the latest technology such as smart speakers, projectors, and internet karaoke.

Regarding the environment of the study, a system was put in place that enabled all staff members to reflect on their daily work activities through the facility's LINE chat tool and to receive AI reports.

The period of the study was 3 months, from July 1 to September 30, 2024.

#### 4.1.7 Overview of participants in the study

An overview of the five elderly care workers who participated in the study is shown in Table 7.

**Table 7:** Overview of elderly care staff in study.

Staff	Age	Elderly care experience	Role	Background
A (Female)		7 years	Administrator (manager/leader), management, guidance, daily care	She has worked at the company since its inception and has participated in community activities.
B (Female)	60s	More than 10 years	Consultant (sub-leader), management, guidance, daily care	Extensive elderly care experience, highly evaluated by users.
C (Male)	60s	6 months	Recreation, daily care	Moved from a computer-related job.
D (Male)		1 year	Recreation, cooking, daily care	Former stage actor with excellent communication skills.
E (Female)	50s	1 year	Recreation, daily care	Bodybuilder, good self-discipline.

The organization in the study is led by two managers with approximately 10 years of experience in elderly care. It consists of two managers and three new staff members with approximately 1 year of experience, who are being trained by the two managers. There are no mid-level staff members.

#### 4.1.8 Ethical considerations

This study was conducted with the approval of the ethics committee of the Japan Advanced Institute of Science and Technology (ethics review number: 人04–038). The elderly care staff who participated in this study were informed in writing of the purpose, methods, and handling of data for the study, and their consent to participate was obtained. All data was anonymized so that individual staff members could not be identified. The data collected is used only for this study.

### 4.2 Results of the study

The results were divided into six periods based on the characteristics of trends in the 12 weeks after the start of the study. The relationship between the fluctuations in the evaluation of each employee in each period and important events was plotted on a line graph. In addition, GTA analysis was conducted on the staff's reflective posts and manager interviews. Furthermore, specific examples of how individual and organizational change was promoted through AI reports and manager guidance are also introduced. First, the transition of evaluation and significant events are shown to visually clarify the change process.

#### 4.2.1 Summary of results during the period of the study

Before the study began, the facility in the study needed to create a manual summarizing the specific work procedures for elderly care. In response, the researcher proposed this method to create a change that would enable the staff to think about appropriate work procedures for each situation in a proactive manner.

As shown in Table 2, the 12-week period of the study was divided into six periods according to characteristics of the period. The period of the study showed a wide variation in motivation to participate in the study in the early stages. Most participants reflected on their work procedures, but their interest shifted to collaboration with others or organizational work after junior high school student volunteers started to participate. During the growth promotion period, the manager confirmed the trend of changes in the content of the reflections, and organizational work was further promoted through onsite guidance tailored to the changes of each staff member. The organization's common goal was recognized as organizational work, and the success of the summer festival, in which each staff member took on a role, and local volunteers also participated, was linked to this change. The recognition of the results of organizational work led to a deepening of interest in others, and we saw an increase in the number of people teaching each other in group chats.

These staff members' independent activities strengthened the organization's cooperation and promoted the sharing of a sense of purpose.

#### 4.2.2 Relationship between individual changes and events

Figure 3 illustrates the weekly evaluation scores and major events across the six phases of the study period. Overall, staff evaluation scores showed an upward trend throughout the six phases. During the initial phase of the reflection introduction, significant improvements were observed in self-initiative and sense of purpose, with staff members becoming more aware of their personal growth.

After the Habituation and Awareness, collaboration among staff members deepened, and organizational work scores improved, further elevating the overall evaluation. Events such as summer festivals enhanced a sense of unity within the organization. When community residents participated in events, staff had opportunities to self-reflect objectively, promoting conscious behavioral improvements. These external stimuli and events served as important triggers for accelerating changes.

The 12 weeks were divided into six phases based on qualitative analysis of staff reflections, identifying key shifts in focus and engagement. These phases, outlined in Table 2, are:

1. Initial Engagement Phase: Primarily focused on work procedures and task execution.
2. Habituation and Awareness: Gradual shift towards awareness of collaboration with others.
3. Learning Enhancement Phase: Increased engagement and emergence of team-oriented reflections, coinciding with the participation of junior high school volunteers.
4. Organizational Cooperation Strengthening (Weeks 7–8): Improvement in organizational work scores as collaboration among staff intensified, especially during joint activities like summer festivals.
5. Organizational Learning Phase (Weeks 9–10): Sustained organizational changes were observed, with improvements in user care quality and peer learning culture emerging in group chats.
6. Result-Sharing Phase (Weeks 11–12): The entire organization strengthened its alignment with the facility's philosophy, and purpose-driven behaviors became habitual, supported by regular AI-generated reflections.

Figure 3 shows each staff member's total scores (maximum 100 points) across these phases. The two senior staff members with higher initial scores demonstrated stable engagement from the outset. Among the three junior staff members with lower initial scores, two showed significant improvements as their focus shifted toward collaboration and leadership, notably coinciding with the participation of junior high school volunteers. Mentoring less experienced individuals fostered stronger collaboration awareness.

One staff member focused on mastering their work procedures, showing no significant increase in evaluation scores within the measured indicators. However, these individuals consistently performed their duties stably and reliably based on established procedures.

Figure 3 shows the total development scores for each staff member, calculated weekly over the 12-week period. These scores are based on the 10 evaluation items in Table 5, each rated on a 10-point scale, with a maximum of 100 points in total. These are quantitative scores and are not related to the qualitative findings from the Grounded Theory Approach (GTA). GTA was used for analyzing reflection content, while the scores in Figure 3 represent numerical growth.

#### 4.2.3 Exploratory observation results on the impact of AI support

AI-generated outputs reduced the time required for managers to grasp the trends in staff records from approximately 45 min (with manual summarization) to about 5 min. In addition, after receiving AI outputs, staff reflections showed an increased frequency of perspective-related terms such as “team collaboration” and “supporting others.”

Furthermore, no significant differences were observed in perspective shifts or summarization tendencies when generating similar summaries using other large language models (LLMs), such as Claude and Grok. This suggests that the outputs of ChatGPT-4o were not unique in this regard.

#### 4.2.4 Weekly and monthly reports generated by AI

##### 4.2.4.1 Weekly report result excerpt

Table 8 shows a specific result excerpt of a weekly report. Based on daily reflection, the weekly report is organized in the format of “date,” “staff,” “review content,” “realization,” and “purpose.” The AI lists this information, and after the manager has checked the content, it is shared throughout the organization. This improves transparency in the workplace and makes it possible to identify and visualize areas for improvement.

**Table 8:** Specific examples of weekly reports (late August: period of enhanced organizational collaboration).

Date	Staff	Reflection contents	Awareness	Objective
Aug. 21 (Mon.)	A	Users actively participate in welcoming situations.	It is important to create an environment where users feel safe.	Promotes user relaxation.
8/23 (Wed.)	D	Devise action plan to support users in situations where users are anxious.	Consideration for the user facilitates the action.	Smooth service delivery.
Fri. 8/25	B	Observe situations where users voluntarily put away dishes.	Support that respects independence is effective.	Supporting the independence of users.

##### 4.2.4.2 Monthly report result excerpt

Table 9 shows a specific result excerpt of a monthly report. Based on the weekly report and AI data analysis, the monthly report visualizes long-term trends and changes in individuals and the organization. This clarifies changes in staff behavior trends, and areas that need improvement and provides a basis for managers to generate personalized guidance plans.

**Table 9:** Specific examples of monthly reports.

Staff	AI generated documents (monthly reports)
Staff B	Staff B's attitude toward psychological understanding of users improved throughout August. Specifically, there were occasions when she observed how users reacted to recreational activities and made suggestions for improvement based on her observations. In particular, they focus on creating an environment that facilitates the active participation of users, and this has improved user satisfaction. In addition, there are many observed situations in which users act spontaneously and actions are taken to respect them. Specifically, there were reports of situations in which users’ independence was drawn out and supported in the cleanup work after lunch. On the other hand, there were also some issues with the lack of opportunities for cooperation with other staff members, and more awareness is needed to strengthen the organizational work.
Staff C	Staff C is becoming more attentive to the well-being of the users throughout August. In situations where users are feeling anxious when bathing, she is flexible and responsive to the situation, and actions that increase the users’ sense of security are reported. In addition, there is a noticeable attitude of support while paying attention to the users’ physical condition and behavior. On the other hand, one issue is the low volume of records of look-backs. This has resulted in situations where managers have not been able to obtain sufficient data to understand specific points of change. The enhancement of the content of the look-backs is an issue for the future.

#### 4.2.5 Individual change and the application of management guidance

Table 10 compares the GTA analysis of the overall review of all staff with the GTA analysis of the manager interview to check the staff situation and shows the characteristics of the perspective of each period. In the initial introduction period, there was an expectation among staff and managers that introducing the review would promote change among staff. In the adaptation period, there was a difference in focus, with staff focusing on improving their work while managers aimed to strengthen cooperation within the organization. However, from the growth promotion period onwards, there was a trend where staff gradually focused on contributing to the organization.

**Table 10:** Results of GTA analysis of staff reflections and manager interviews for each period.

Period	Reflection of all staff	Interview with the manager	Direction
Initial stage of introduction	Increased independence and a proactive approach to work appear.	Expect changes in staff through reflection.	Match
Adaptation period	Reflection creates an awareness of the need to improve operational efficiency and quality of care.	Cooperation among staff is strengthened and organizational unity is advanced.	Employees are individually-oriented, managers are organizationally-oriented
Growth spurt	Organizational work will be strengthened and cooperation among staff will be established.	Recognition of the need to adapt guidance to the changing needs of individual staff members	Staff is organizationally-oriented, managers are individually-oriented
Period for strengthening organizational cooperation	Motivation and a sense of cooperation will increase through event activities.	Recognition of staff autonomy and leadership.	Match
Organizational learning period	Improvements in daily operations have been made, and users’ independence support has been enhanced.	We expect that changes in staff will contribute to the improvement of the organization as a whole.	Match
Result-sharing period	Operations have been facilitated by the establishment of a cooperative system through review.	The staff and the organization's cooperation is valued as it supports the growth of the organization as a whole.	Match

#### 4.2.6 Changes achieved by staff member D in the cooking area

Table 11 presents how Staff Member D demonstrated growth during the study. While the overall learning cycle (reflection → AI analysis → managerial guidance → behavioral change) supported this process, the focus here is on specific behavioral changes observed in work efficiency improvement.

**Table 11:** Examples of management guidance for staff D.

(Data) item	Self-reflection	AI analysis	Supervisory guidance	Change
Efforts to improve operational efficiency	Focused on improving food delivery calculations and serving procedures, and documented reflections on work efficiency.	AI provides feedback on the usefulness of meal preparation techniques (e.g., food prep) to improve efficiency.	The manager explained to D that failure is part of the learning process and advises D to organize the dishes and utensils efficiently.	Increased work efficiency through improved meal preparation.
Strengthen health and safety management	Focus on sanitation and user safety.	The AI assessed that sanitation is important for user health and confidence building.	Guidance on cleaning, food control, and sanitizing utensils.	Gaining the trust of users by strengthening sanitation management.
Improved communication	Focus on sharing roles and information with other staff and users.	AI noted that cooperation is essential for organizational work and suggested strengthening the division of roles.	Guidance on clarifying roles and improving communication methods.	Smoother organizational collaboration.
The challenge of new approaches	They were challenged to come up with new cooking methods and menu ideas, and the results were recorded in their reflections.	AI evaluated that the introduction of the new methodology contributed to increased user satisfaction.	Advice on adjusting menus to user preferences and improving cooking.	The introduction of new methods brightened the workplace and improved user satisfaction.

For example, Staff D initially documented daily reflections centered on improving the calculation and procedures for food service. The AI monthly report identified these efforts as consistent trends and highlighted their potential contribution to improving work efficiency. Based on this, the manager provided targeted guidance, such as suggesting ways to optimize workflow and delegate tasks related to food service operations.

As a result, Staff D implemented procedural changes, which were later reflected in both the evaluation scores (especially in problem-solving ability and organizational work) and the content of subsequent reflections, where Staff D reported improved time management and collaboration with colleagues in food service delivery.

#### 4.2.7 Specific example of a goal-oriented manual that incorporates organizational knowledge and reflection

Table 12 shows the process by which AI analyzed the knowledge gained from Staff Member D's daily reflection, and a goal-oriented manual was generated that was used to improve operations through the guidance of managers. In this table, the roles of users, staff members, counselors, and managers are arranged along the horizontal axis, and the flow of operations is expressed along the vertical axis by the time axis. As an initiative for supporting users' independence, users were asked to participate in cooking and serving food to the extent possible.

**Table 12:** Purpose-oriented manual reflecting culinary knowledge from reflection by staff D.

Role/order	User (elderly)	work procedure (based on D's reflections)	Purpose of the work (based on D's reflections)	Vision of the work (AI generated)
10:00	Checking the freshness of ingredients	Check ingredients and sanitation	Provide safe and clean ingredients to users.	Focus on culinary hygiene and user safety
10:15		Preparation of distribution calculations, calculation of quantities of each component	Adjust based on nutrient requirements for each user.	Appropriate distribution to each user
10:40		Efficient cooking procedures	Aiming for smooth service by improving work efficiency.	Ensure efficient and accurate cooking
11:00		Begin preparing a variety of foods (main and side dishes, etc.)	Adjust to the user's preferences and health status.	Consider user preferences and health
11:40	Participate in cooking or serving food	Identify the division of roles among staff members.	Facilitate organizational work to ensure smooth workflow.	Encourage integration of food preparation and serving
11:45		Taste each dish to confirm seasoning.	Consider the health status and dietary preferences of the user.	Focus on improving satisfaction through cooking
12:00	Wait for food service	Provide accurate portions of meals.	Provide safe and balanced meals to users.	Ensure an appropriate eating environment for users

Staff Member D's reflection included specific insights regarding improvements in “reviewing work procedures,” “user health and safety management,” and “role sharing within the organization.” AI organized and analyzed these contents and applied them to the guidance provided by the manager. As a result, specific changes were made to actual operations, including improvements in Staff Member D's work efficiency, adopting cooking procedures that take into consideration user participation, and strengthening cooperation between staff.

#### 4.2.8 Applying organizational knowledge and organizational work

While no direct quantitative comparison with previous events (e.g., Christmas festivals) was available, past challenges in staff cooperation were noted through manager interviews. The observed improvements in cooperation during the summer festival reflect the organizational learning accumulated during the study period.

Table 13 shows that the accumulation of learning from daily work activities resulted in increased cooperation at the summer festival. The changes in work attitude/behavior of each staff member led to cooperation at the summer festival event. The individual learning and insights gained through daily work activities were shared through reflection activities and accumulated as organizational knowledge. This knowledge accumulation helped strengthen cooperation and collaboration between staff members and was applied to their respective work responsibilities.

**Table 13:** Staff responsibilities and objectives and organizational work at summer festivals.

Staff	Areas of responsibility	Roles and specific activities	Objective	Organizational work
A	General operation	Responsible for coordinating the overall progress of the event and working with other staff and volunteers to manage the cooking and venue set-up to ensure smooth operation	Ensure the smooth running of the entire event and strengthen staff coordination.	Strengthen the partnership between staff and volunteers and serve as the overall coordinator.
B	Food and beverage booth	Focus on food management and food delivery to ensure that participants can enjoy the event safely.	Ensure the safety of meals and provide a comfortable environment for users to eat.	Coordinate and cooperate with other staff members to facilitate the timing of meal service.
c	Game booth	Guiding and assisting participants and interacting with users	To provide enjoyment and safety to users and encourage them to participate in activities.	Work with other staff to create a fun atmosphere.
D	Information desk	Responsible for guiding and supporting participants to the venue and watching over the elderly.	Provide users and participants with a sense of security and support smooth event operation.	Share information with other staff to support smooth operations.
E	Facilities management	Procure necessary equipment for the event and assist other members in facilitating the event.	Promptly arrange for necessary equipment and materials to support event operations.	Work with all staff to ensure necessary equipment and support.
A	General operation	Responsible for coordinating the overall progress of the event and working with other staff and volunteers to manage the cooking and venue set-up to ensure smooth operation	Ensure the smooth running of the entire event and strengthen staff coordination.	Strengthen the partnership between staff and volunteers and serve as the overall coordinator.

Each staff member has a different role at events like the summer festival, but the staff members must cooperate towards achieving a common goal (i.e., success of the summer festival).

#### 4.2.9 Feedback from participants 1 month later

Table 14 shows the results of interviews with participants 1 month after the study ended and is divided into four categories: “documentation,” “ individual knowledge,” “development,” and “organizational knowledge.” Table 14 summarizes participant feedback 1 month after the study, categorized into four areas: documentation, individual knowledge, development, and organizational knowledge.

• Documentation: Participants found that recording reflections helped clarify their thoughts and supported ongoing self-awareness.
• Individual Knowledge: Many reported new insights and perspective shifts, especially when comparing their reflections.
• Development: Staff described improved collaboration and work efficiency, supported by AI feedback offering different viewpoints.
• Organizational Knowledge: Participants noted that knowledge sharing and teamwork improved, with reflections contributing to organizational learning.

**Table 14:** Participants’ feedback after 1 month.

(Data) item	Speaker	Statement	Interpretation
Recording	A	Even those who were obliged to do so were able to think about the content.	Recording the findings will increase awareness of business improvement and enable purposeful action.
	D	Sometimes people forget what they have even noticed. That is why it is important to take notes of what you notice.	By taking notes, you can record your findings and use them to reflect on and improve your work.
Personalization of knowledge	A	When the purpose is clear, the work becomes meaningful and the quality of the action increases.	Through notes and reflection, work can be performed with an awareness of purpose and the quality of actions can be improved.
	c	The thing is that notes are a very effective means of review. Review is more effective than preparation in terms of deepening understanding of the work.	The use of notes as a review is effective in consolidating learning.
Development	A	By considering the purpose, the work becomes more than just routine.	Through reflection activities, the participants gain a sense of purpose and improve their behavior.
	c	Forcing yourself to find and record one awareness will lead to development.	The habit of recording, which is done with great effort, leads to the discovery of new learning and development.
Organizational knowledge	A	Shared objectives will enable the entire organization to work together.	Shared objectives strengthen organizational work and organizational unity.
	B	Consultation became closer and communication easier.	Strengthening collaboration through reflection activities promotes cooperation among staff members.
	C	By using memos to share objectives, the answer to the question, This day service has these values.	Sharing objectives through memos contributes to a sense of unity throughout the organization and unification of work direction.

In summary, the feedback suggests that shared reflections strengthened individual learning and that the combination of self-reflection, AI analysis, and managerial guidance supported both personal and organizational growth. The specific comments made by each participant are shown in Table 14.

## 5 Discussion

### 5.1 Individual reflection

Staff development involves reflecting on day-to-day work activities and increasing self-awareness. This process of reflection can be explained using Kolb's experiential learning theory and the S-ART theory (Self-awareness, Self-regulation, Transcendence). Kolb's experiential learning states that learning is a cyclical process of “concrete experience,” “reflective observation,” “abstract conceptualization,” and “active practice” through which personal experience is internalized (10). This study confirmed that the insights gained through reflection lead to purpose-oriented behavior and self-improvement and that staff members understand “why they are taking this action,” which is reflected in their reflection on purposeful behavior rather than simple action without reflection. In the case of daycare services, the daily work routine is repetitive, from welcoming users to seeing them off. Depending on the day's situation, staff members can repeatedly discover the meaning and methods of their actions in the repetitive similar actions. It is an environment that makes it easier to discover new insights and promotes improving capabilities. Furthermore, cloud tools such as LINE groups make it possible to flexibly view and write reflections of oneself and others without limitations on time or place, and it is very effective for individual improvement efforts.

Through the S-ART theory, Vago and Silbersweig (22) also suggest that self-awareness and self-regulation contribute to psychological growth and an improvement in the awareness of goal achievement. This theory explains that deepening self-awareness leads to behavioral adjustment and personal growth that harmonizes with others and the organization. Based on this theory, reflection supports staff growth by following the process of “making the purpose conscious” and then going through the processes of “deepening self-awareness,” “increasing autonomy,” and “improving behavior” as described in this theory. First, by making the purpose conscious, staff can clarify their work's “purpose” and “behavior.” Next, as the sense of purpose increases, they will understand their current capabilities and behavior more deeply, and their autonomy will increase, leading to positive behavior improvement. This process leads from self-awareness in S-ART theory to self-regulation and ultimately to transcendence, which contributes to the organization's overall results.

From the perspective of both Kolb's experiential learning theory and S-ART theory, reflection is essential for staff members to improve their self-awareness and behavior. In particular, by sharing documented reflections, staff can improve their behavior with a sense of purpose and achieve results for the organization. As we saw in the summer festival event, this leads to a sense of accomplishment and motivation for the next step. Therefore, it is important to strengthen the mechanisms that support this process to prevent a decline in staff motivation to provide physically and emotionally demanding services such as elderly care.

### 5.2 The role of AI

The AI used in this study (ChatGPT-4o) processes vocabulary, syntax, and perspective expressions in reflection records to generate summaries that capture changes in self-reflection and learning depth. Specifically, shifts in perspective (from self to others and the team) and frequent use of introspective phrases (e.g., “noticed that,” “thought that… is necessary”) were reflected in the monthly reports. This suggests that AI-driven language processing can help visualize staff development over time. However, the AI's output is based on statistical inference, so its processing remains partially opaque. Therefore, this study attempts to clarify the linguistic features underlying AI outputs and connect them to observations of human learning behaviors.

While AI can process reflective records and highlight learning trends, these insights need to be integrated into practice. This is where the collaboration with managers becomes crucial. AI is important in enhancing the staff reflection process and elevating it to learning for the entire organization. However, this cannot be achieved by AI alone and is maximized through manager collaboration. In this respect, the objective analysis and structuring of knowledge by AI and the judgment and guidance of managers can function in a mutually complementary manner.

By analyzing reflective data and objectively visualizing areas for improvement and development trends, AI provides a foundation that enables staff members to accurately evaluate their behavior and plan their next course of action. For example, as can be seen in the results of the study in Table 11: Example of manager guidance for Staff Member D, suggestions such as “work efficiency improvement” and “communication improvement” provide the basis for managers to provide specific guidance tailored to each staff member's situation.

This process supports the effectiveness of the data-driven learning proposed by Ajoodha et al. (23), and the collaboration between AI and managers promotes staff self-reflection and development. Furthermore, AI analyzes individual staff member's reflections and systematizes the data obtained, contributing to the accumulation of organizational knowledge. This process of structuring knowledge is based on Ijuin's (19) “purpose-oriented knowledge structuring,” and it supports knowledge sharing and behavior improvement by structuring the reflection data in relation to the purpose of the action. More specifically, the weekly report generated by AI lists the individual reflection data, allowing managers to understand the overall issues and points for improvement. Furthermore, in the monthly report, the AI clarifies midterm growth trends and changes based on the weekly results of each individual, and managers can use this information to provide guidance, which is then shared onsite. This process is based on knowledge management theory (25) and is linked to the purpose-oriented knowledge structuring proposed by Ijuin (19). The input of the vast amount of reflective data in this process, the creation of summaries and lists that accompany this data, and subsequent content analysis are tasks that are beyond the capabilities of human labor and are processes that can only be carried out by AI. The results of this study have shown that this is what helped the managers provide appropriate staff guidance.

The roles of AI and managers are not just a matter of division of labor but complementary. By having AI perform data-driven objective analysis and managers providing flexible guidance based on the results, the efficiency and results of the entire organization can be improved. This collaboration is essential in enhancing the organization's learning capacity and is the foundation that supports the growth of individuals, groups, and the entire organization (26).

#### 5.2.1 Consideration of exploratory observations on the impact of AI support

Although this study did not employ a controlled experimental design, exploratory observations suggested that AI-generated summaries contributed to qualitative changes in staff reflection, such as shifts in perspective and a clearer sense of purpose. Similar trends in perspective were also observed in outputs generated by other large language models (LLMs), such as Claude and Grok. This suggests that the output of ChatGPT-4o may not be a result of model-specific bias but may instead reflect a certain level of reproducibility.

### 5.3 Skills required for managers

In the AI-based reflection process, managers support staff development and contribute to the organization's overall efficiency. Managers need appropriate skills to effectively use AI reports.

First, managers need to be able to accurately understand the reports generated by AI. To do this, managers need to understand AI reports strictly according to their role and consciously eliminate any bias or prejudice towards individual staff members. At the same time, it is also important to maintain objectivity towards AI and not become too dependent on it. Furthermore, it is the role of managers to transform this information into a concrete action plan and communicate it to staff members.

Next, for staff members to accept the AI reports and actively work to improve their behavior, it is essential to ensure psychological security. Managers are responsible for creating an environment where staff can share learning without fear of failure. Managers need to have a positive attitude, avoid negatively receiving the content of the reports, and adopt the idea that “failure is part of growth” as part of the workplace culture. The results of this study showed this in the managers' guidance in the cooking events in Table 11.

In addition, managers can be more effective in motivating staff by adding their insights and thoughts according to the actual situation to the AI reports. As shown in the results of this study, “On the other hand, the small amount of reflection recorded is an issue.” In Table 9, “Specific examples of monthly reports,” the analysis results shown by the AI may sometimes seem automated and cold.

As we have seen, managers need to be able to “understand and utilize AI reports.” They also need to be able to “ensure psychological security of staff” so that they can accept the AI's findings, which arise from the facts, in a way that is easy to accept depending on the situation and the characteristics of the staff. They need to have the “ability to support individual development.” When managers can demonstrate these capabilities, they can supplement the limitations of AI and contribute to the development of staff and the efficiency of the organization as a whole. Managers can fulfill their role as a “bridge” between AI and staff. In the future, developing training and support systems to help managers acquire these skills will be essential.

### 5.4 Organizational learning and organizational change

**Figure 4:**
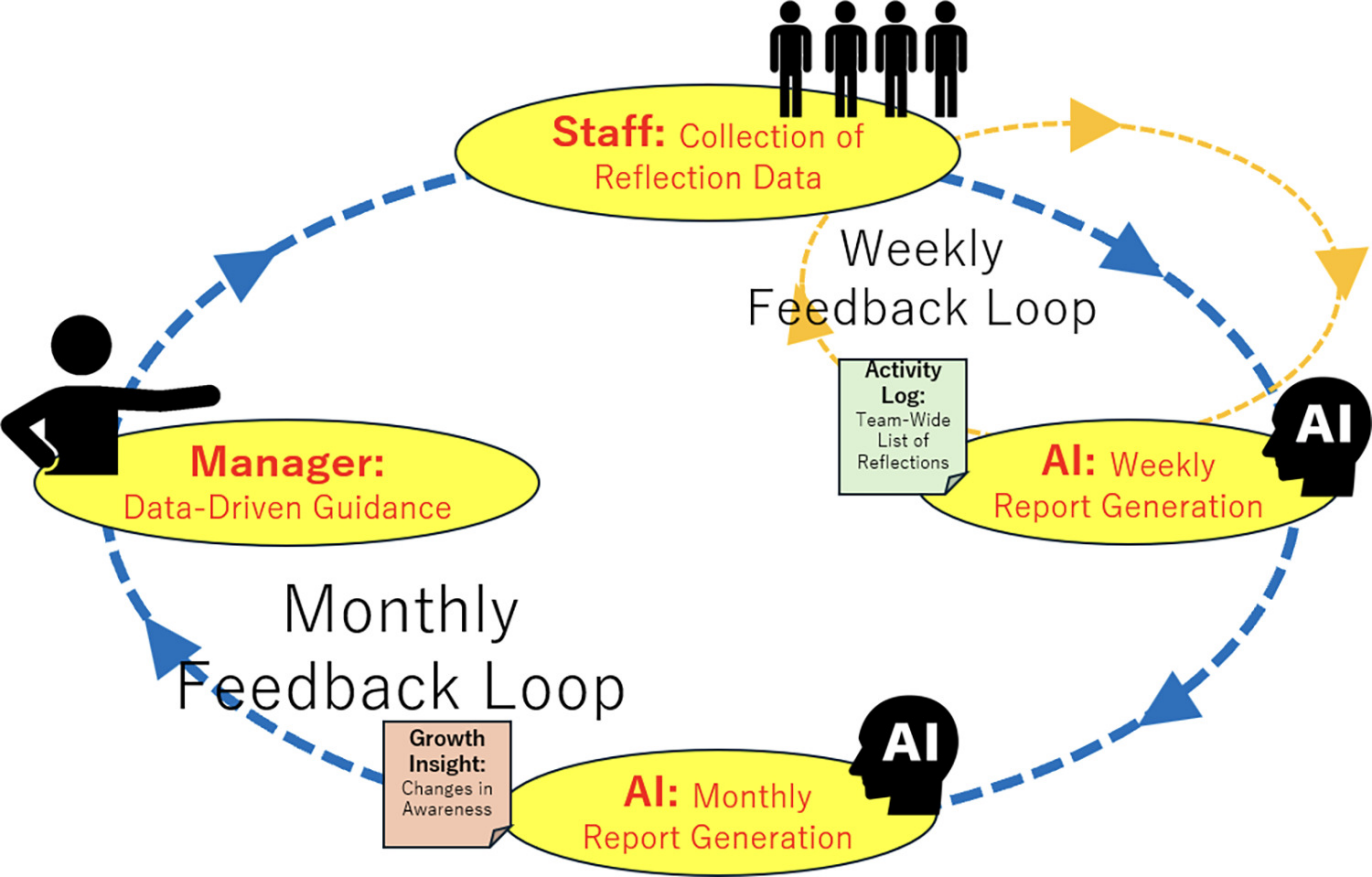
Weekly and monthly feedback loop.

The process of staff reflecting on their work and identifying areas for improvement is an important foundation for organizational learning as a whole. The “feedback loop” in Figure 4, used in this study, refers to a system in which staff reflection and learning are shared within the organization weekly and monthly and then further deepened through AI analysis and management guidance. This loop is a structure for individual learning to be fed into the organization.

The AI system collates and lists the data from each staff member's reflection and then produces a weekly report. Every month, the manager uses the analysis results from the weekly reports to formulate medium- to long-term goals and improvement plans and provides guidance to indicate the organization's overall direction. In this way, this loop forms the central mechanism that links each staff member's development to the organization's overall results.

From the perspective of organizational learning proposed by Senge (8), it is clear that this feedback loop also has a mechanism in place that links individual growth to the growth of the organization as a whole, mainly through the sharing of goals and objectives by members of the organization.

In addition, based on the S-ART theory (22), as staff members increased their self-awareness, their empathy and sense of cooperation with others increased, which led to stronger collaboration within the organization. This theory supports the process by which, with the support of AI and managers, self-reflection develops into social learning that transcends the individual.

Furthermore, as shown in Table 10, “GTA Analysis Results of Staff Reflection and Manager Interviews for Each Period,” during the adaptation period of the reflection activities, staff members focused on individual reflection, while managers focused on organizational collaboration. However, from the growth promotion period onwards, staff members gradually became more aware of contributing to the organization as a whole, and their way of thinking became more aligned with the guidance policy of the managers. This shows that the guidance of the managers contributed to the organization as a whole moving in the direction of collaboration and the improvement of overall results. This process is also consistent with the formation process of the “learning organization” proposed by Senge (8). It shows a cyclical mechanism between individual reflection and collaboration across the whole organization.

The event in this case study, “Table 13: Staff Responsibilities, Objectives, and Organizational Work at the Summer Festival”, demonstrated how individual development led to organizational results and how each staff member voluntarily took on a role and cooperated with a sense of purpose, strengthening the organization's unity. In this process, by using the data provided by AI and the guidance of the manager, the staff members had the opportunity to recognize their own roles and understand how their individual actions affect the organization's overall results. In this way, by sharing goals, harmony was achieved throughout the organization, and as a result, it became possible to provide high-quality services to the local community.

The knowledge and experience shared throughout the organization is not just a collection of information but stored in the organization's knowledge base. This knowledge base serves as a foundation for solving future issues and new projects and as an element that strengthens the organization's collaborative nature. In this way, by working together, AI and managers have created a system in which learning that begins with individual reflection is returned to the entire organization and sublimated into the growth of the entire organization.

Summarizing the above, a “new AI-based reflection support method” has been developed that aims to improve the entire organization's results by strengthening individual development and organizational collaboration.

### 5.5 Cross-checking against the interviews

According to Table 14, “Participants” feedback 1 month later, the participating staff members understood the importance of personal learning and the reality of professional development. They understood through their own experience that personal development led to organizational development. Even the staff members who were initially less motivated to participate in the study changed their behavior so that learning would be effective as they became aware of their development, leading to further personal and organizational development.

During an interview, Participant D said, “The best thing is to record it when you realize it,” emphasizing the importance of recording your realizations immediately. This behavior effectively promoted the subsequent “reflective observation” and “abstract conceptualization” by leaving a record of the “concrete experience” as described in Kolb's theory. This immediacy enhances the quality of learning and makes more effective use of experience.

In addition, the comment “finding and recording even one insight, even if it is forced, leads to growth” supports the self-awareness and self-regulation of the S-ART theory (22). Recording insights and repeating this process leads to the development of habits, and the process of improving work performance and personal development is shown.

Furthermore, the comment “Using memos to share our goals has helped us to clarify our direction” shows that the personal records spread throughout the organization. The results support Senge's (8) theory of organizational learning. It was clear that personal learning promoted the sharing of organizational values and direction and contributed to achieving the organization's overall goals.

All of these comments show that the staff, who initially waited for instructions regarding their work, became proactive through the study and actively involved in improving their work and sharing knowledge. It was confirmed that recording and accumulating individual insights led to the organization's growth. The findings of this study are considered to apply not only to elderly care facilities but also to the improvement of knowledge management and organizational learning in other fields.

Although these results were obtained through a small-scale case study, they demonstrate the effectiveness of this growth promotion method as an easy-to-introduce technique with a high impact on small—to medium-sized elderly care facilities.

### 5.6 Possibility of application to other fields

The AI-assisted reflection process proposed in this study was shown to be effective in reducing workload in elderly care settings and strengthening cooperation between staff members. This method is considered to have high applicability not only in elderly care but also in the following fields.

First, in education, this method can be applied to post-lesson reflection meetings and the sharing of effective teaching methods conducted by teachers. AI is expected to present points for improvement in lesson content and promote efficient knowledge sharing among teachers.

In the medical field, AI can be used to review patient treatment progress and adjust treatment plans. Specifically, AI can analyze patient activity records and progress data, and a system can be built to automatically generate reference materials for staff to use when formulating the next treatment plan.

In addition, AI could be used in the hospitality industry to improve employees’ customer service skills and the customer response process. For example, AI could analyze records of employees' customer service activities and build a system that makes specific suggestions for improving customer satisfaction. This would enable employees to share good customer service methods, making it possible to improve the efficiency of new employee training and the quality of service.

Furthermore, it is also possible to apply this method of making suggestions to users of daycare services. More specifically, by collecting information on users' daily activities and any changes in their physical condition, as well as the background to these, in a group chat and having AI analyze this, it is possible to build a system that automatically generates reference materials for use when generating subsequent care plans. For example, suppose a record shows that the user feels fatigued after afternoon activities. In this case, AI can use data such as the amount of food consumed and the amount of sleep to determine the cause and then suggest appropriate meal plans and rest periods to maintain energy levels. In addition, when instability during walking is observed, the system can analyze past rehabilitation records and activity levels to support the adjustment of appropriate exercise programs and rehabilitation plans. By utilizing AI analysis in this way, it is possible to provide personalized care according to the user's physical condition, and this not only contributes to improving the quality of care but also enables the sharing of information between staff and streamlining the care process. Furthermore, based on the insights gained, it is possible to strengthen cooperation between family members and medical professionals, which can improve the overall support system of the user.

Further field tests and data collection under different conditions are necessary to demonstrate the applicability of this method in other fields. We will conduct these tests as the next research topic to clarify that this method can be generalized and applied in a wide range of fields.

## 6 Issues and prospects

In this study, we proposed a method to support reflection activities through collaboration between AI and managers, improve staff behavior, and strengthen organizational collaboration. However, some issues remain in the current state, and overcoming these issues will enable further application.

### 6.1 Issues and limitations

At present, the structure of AI-generated outputs can only be inferred, as there are no means to fully verify the model's internal processes. In this study, explicit analytical methods such as sentiment scoring or clustering were not applied, so the visualization of emotional tendencies remains limited.

Future work should focus on developing quantitative indicators for perspective shifts and levels of reflection, allowing for validation against AI outputs. Additionally, introducing sentiment analysis and visualization techniques will help clarify the relationship between the components of LLM-generated summaries and organizational learning.

To better assess the causal effects of AI-assisted summarization, controlled experimental designs—such as pre-post comparisons and the inclusion of control groups—are needed. Furthermore, for managers to interpret and utilize AI outputs appropriately, training programs and operational guidelines should be developed to ensure sound decision-making. Continuous comparison with outputs from different LLMs is also essential to enhance the reliability and generalizability of AI-generated summaries.

Current AI tools are effective in analyzing and structuring reflection data, but the accuracy of the reports generated depends on the quality of the data entered. For example, if the reflection is written ambiguously, the analysis results shown by the AI will also lack specificity. To address this issue, it is important to support the user in a way that leads to a sense of purpose while respecting free-form input. Recording the most memorable event in the daily reflection can deepen the staff's awareness and gain new insights and hints for improvement from unexpected inputs. Another issue is that AI cannot wholly supplement flexible judgments in response to environmental factors and site-specific constraints. In this regard, it is necessary to set up a place where managers and staff members can consider solutions together using their knowledge of the site and the AI analysis results.

In addition, the study was limited to five elderly staff members, and the small sample size may have affected the generalizability of the results. Furthermore, the period of the study was short, 3 months, and the long-term impact and sustainability of the proposed method could not be verified. Furthermore, the AI tool used depended on ChatGPT4.0, so it is unknown whether similar results could be obtained using other technologies.

In the future, to generalize the method, large-scale surveys across multiple facilities and long-term follow-up studies with an increased sample size will be necessary. Validation using AI tools other than ChatGPT 4.0 will also be required.

Furthermore, there are differences in the ability of managers to utilize the reports generated by AI in the workplace, and in particular, there are indications of a lack of skills related to psychological security, such as “independence” and “empathy.” For example, if there is a lack of ability to effectively convey the analysis results presented by AI to staff members and promote dialogue, the results of reflection activities will not be fully demonstrated. To solve this problem, it is necessary to train managers on communication and teaching methods and improve the skills for motivating staff.

Another issue is that reflection activities can create a sense of competition and stress. It is important to foster a culture that allows for failure and can share learning, but this takes time and appropriate intervention from managers. For example, if the results of the reflection are limited to evaluation and criticism, there is a risk that staff motivation will decline. In order to foster this culture, it is effective for managers to view staff efforts and development positively, provide constructive feedback, and regularly create opportunities for everyone to evaluate successful cases and lessons learned.

In facilities without adequate IT infrastructure, barriers to AI implementation include cost, human resources, and digital literacy gaps. At the research facility, we leveraged existing tools widely used in Japan, such as LINE Group, and facilitated a smooth implementation through staff briefings and support from AI-savvy staff. Automating operations and simplifying the user interface will be key for future adoption.

Reflection records include individual perceptions and emotions, so several considerations were made. Records were anonymized to prevent individual identification, and it was clearly stated that AI outputs would not be used for evaluation or assessment. Additionally, managers constantly review and adjust AI outputs before use to prevent overreliance on AI.

This study is a case study involving one facility and five staff members, so there are limitations to generalizing the results. However, clear trends were observed in the quality of reflection and changes in perspective. Conditions such as trust among staff, leadership coordination, and a culture of dialogue facilitated by AI outputs are important for future expansion, and these have been confirmed in this facility. When expanding to other facilities, it will be necessary to establish these conditions.

### 6.2 Prospects

In order to address the current issues regarding AI tools, it is necessary to have a system in place that supports improving the quality of reflective data and its flexible use. In order to enable staff members to record reflective data with an awareness of the “purpose” of their work, it is necessary to establish a system and guidelines that share the knowledge management and underlying goal-oriented thinking with staff. For example, by providing workshops that visualize the relationship between goals and work or simple question formats that reflect an awareness of purpose in daily records, it is likely that staff will be able to make it a habit to record data with an awareness of purpose.

By recording the most memorable event of the day, it is hoped that staff will be encouraged to reflect on their experiences and make discoveries. By using this free-form writing while also providing a question format to aid awareness of purpose and specific examples of what to write, the potential for AI to provide more practical and specific analysis results will be improved.

In addition, in order for AI to be able to make flexible decisions in response to environmental factors and site-specific constraints, it is necessary to have a process where managers and staff participate together in a “solution study group” or similar forum and make decisions by combining AI analysis results with onsite knowledge. Through such collaborative forums, it is possible to make improvements customized to the site and compensate for the limitations of AI.

Furthermore, to eliminate the differences in ability between managers, it is necessary to enhance the educational content that can be understood and put into practice in addition to the training program for managers. This will enable effective communication of AI analysis results to staff and improve the skills needed to motivate them through goal-oriented dialogue. In particular, strengthening skills related to psychological security, such as “independence” and “empathy,” will enhance the sense of unity throughout the organization and lead to better results.

Managers must positively evaluate staff efforts and create opportunities to share success stories and growth to alleviate the competitive consciousness and stress of reflection activities and foster a culture of shared learning. This will create a psychologically secure environment where people can learn from their mistakes and participate in reflection activities with confidence.

To address the fact that AI analysis is biased towards short-term data, it is necessary to expand the functions that visualize long-term trends and the growth of the organization as a whole. To achieve this, it is possible to introduce a system that uses RDB (relational database) and other methods to store reflective data by period and to re-analyze it using AI as necessary. This system allows AI to accumulate and analyze weekly data and generate reports showing monthly and yearly progress, making it easier for managers and staff to understand long-term results. This, in turn, is expected to support the organization's growth as a whole and strategic decision-making.

Finally, because the scale of the study was small and the period short, further long-term empirical research is needed at multiple facilities and offices. Even after the study's completion, reflective activities continued voluntarily, showing expansion as younger generations, including part-time university students, participated. Furthermore, the adoption of this method has begun in other industries, such as other caregiving providers and the education sector, expanding the field to demonstrate the effectiveness and versatility of the proposed method.

## 7 Conclusion

In this study, we proposed supporting staff reflection activities through collaboration between AI and managers, improving behavior, and strengthening organizational collaboration through goal-oriented knowledge. This method has been used to create a system that aims to improve the efficiency and quality of work and has achieved the following results.

By clearly raising staff awareness of the “purpose” of their work, it has become possible to respond with greater flexibility. Staff members were able to choose different methods to achieve their goals depending on the situation. As a result, staff members were able to improve their problem-solving skills and develop a greater awareness of the need for work improvement. In addition, through individual guidance provided by managers based on AI-generated reports, specific improvement measures were proposed for each staff member's issues, leading to effective work improvement. As a result, it was shown that reflection activities improve the quality of individual actions and increase the efficiency of overall work.

In addition, the fact that the organization could share its goals and strengthen its collaborative capabilities was also an important outcome. The sharing of weekly reports, which listed the individual insights gained, and the sharing of knowledge based on the purpose-oriented manual confirmed that the staff was approaching their work more purposefully. This process not only increased the sense of collaboration among staff and improved their problem-solving abilities but also strengthened the organization's sense of unity and cooperative structure.

Furthermore, the staff members who took part in the study themselves stated that they felt that they and the organization had grown through the procedures of this study.

Looking forward, further improvements in the accuracy of AI analysis are necessary. For example, it is possible to consider a system that accumulates long-term data to visualize trends in change and then uses AI to re-analyze the data to identify areas for medium to long-term improvement. It is also necessary to develop training and education programs to help managers effectively utilize purpose-oriented knowledge and improve their skills in sharing knowledge with staff. This will require a multifaceted approach that includes communication skills and teaching methods.

Through these initiatives, it is hoped that a system based on collaboration between AI and managers will be applied in a broader range of workplaces and that individual development and overall organizational performance will be integrated. This approach has the potential to become a model case for achieving sustainable organizational management in a range of fields in the future.

## Data Availability

The datasets generated and/or analyzed during the current study are not publicly available due to the inclusion of sensitive personal data from elderly care settings. Ethical considerations and institutional review board policies restrict public sharing. However, de-identified excerpts may be made available upon reasonable request and subject to ethical approval. Requests to access the datasets should be directed to the corresponding author.
